# Breeding Experience and the Heritability of Female Mate Choice in Collared Flycatchers

**DOI:** 10.1371/journal.pone.0013855

**Published:** 2010-11-04

**Authors:** Gergely Hegyi, Márton Herényi, Alastair J. Wilson, László Zsolt Garamszegi, Balázs Rosivall, Marcel Eens, János Török

**Affiliations:** 1 Behavioural Ecology Group, Department of Systematic Zoology and Ecology, Eötvös Loránd University, Budapest, Hungary; 2 Institute of Evolutionary Biology, School of Biological Sciences, University of Edinburgh, Edinburgh, United Kingdom; 3 Department of Evolutionary Ecology, Estación Biológica de Doñana-CSIC, Sevilla, Spain; 4 Department of Biology, University of Antwerp, Wilrijk, Belgium; University of Bristol, United Kingdom

## Abstract

**Background:**

Heritability in mate preferences is assumed by models of sexual selection, and preference evolution may contribute to adaptation to changing environments. However, mate preference is difficult to measure in natural populations as detailed data on mate availability and mate sampling are usually missing. Often the only available information is the ornamentation of the actual mate. The single long-term quantitative genetic study of a wild population found low heritability in female mate ornamentation in Swedish collared flycatchers. One potentially important cause of low heritability in mate ornamentation at the population level is reduced mate preference expression among inexperienced individuals.

**Methodology/Principal Findings:**

Applying animal model analyses to 21 years of data from a Hungarian collared flycatcher population, we found that additive genetic variance was 50 percent and significant for ornament expression in males, but less than 5 percent and non-significant for mate ornamentation treated as a female trait. Female breeding experience predicted breeding date and clutch size, but mate ornamentation and its variance components were unrelated to experience. Although we detected significant area and year effects on mate ornamentation, more than 85 percent of variance in this trait remained unexplained. Moreover, the effects of area and year on mate ornamentation were also highly positively correlated between inexperienced and experienced females, thereby acting to remove difference between the two groups.

**Conclusions/Significance:**

The low heritability of mate ornamentation was apparently not explained by the presence of inexperienced individuals. Our results further indicate that the expression of mate ornamentation is dominated by temporal and spatial constraints and unmeasured background factors. Future studies should reduce unexplained variance or use alternative measures of mate preference. The heritability of mate preference in the wild remains a principal but unresolved question in evolutionary ecology.

## Introduction

High genetic variability is common in sexually selected traits [1]. The mechanisms that generate and maintain this variation [2] include the evolution of variance-increasing genetic mechanisms [1], bias in receiver attention towards more variable characters [3), and the evolution of condition-dependence in costly signals [4] with the concomitant enrichment of trait genetic variance by the genetic background of body condition [5], [6]. Individual variability in mate preferences, on the other hand, is less widely appreciated. Mate choice usually exerts a directional selection pressure on signal traits [7] which may lead to morphological evolution [8], population divergence [9], [10] and speciation [11]. However, mate choice also exerts directional selection on male attributes indicated by the signal trait that confer high fitness in the given environment, thereby facilitating adaptation to the prevailing environment [12], [13]. Due to the evolutionary importance of directional selection pressures imposed by mate choice, mate preferences are often considered as attributes of the given population. For example, it is a common practice to assay overall selection on the signal trait via pairing success, and to compare it among populations of the same species [14], [15], [16].

However, directional mate choice also shows variation within a single population. For example, females that are unattractive or in poor condition may need to be less choosy due to the risk of rejection by males [17] or the costs of mate search [18]. Alternatively, these females may exercise stronger mate choice to compensate for their own handicap [19]. If there are trade-offs between different heritable and non-heritable male traits of interest to females (e.g. parental contribution versus good genes), females may need to balance between the competing needs [20], [21] and may therefore diverge in their preferences [20], [22]. But while individual preference may therefore vary, is this variation heritable?

Theoretical models that laid the foundation of contemporary sexual selection research examined the evolution of mating preferences, which means that they assumed heritable variation in preferences (reviewed by [23]). In the case of low preference heritability, classical theories of sexual selection would largely lose their applicability. Both the Fisher process and the good genes model envisage a genetic link between (typically female) preference and (typically male) ornament expression. This link, in the form of a genetic correlation, cannot exist if preference (or ornament) is fixed and non-heritable [24]. Genetically fixed preference for traits indicating genetically based offspring condition would work only if the genetic basis of general body condition contributed to variation in the ornament [i.e. condition-dependence, 6]. On the other hand, fixed preference for ornaments indicating specific types of condition-related benefits (including direct benefits, [25]) may not be adaptive in the long-term, given the general observation that fluctuating selection on these fitness-related traits is common in nature [26]. Finally, lack of additive genetic variation in female preference would also make it more difficult for new ornaments to evolve [27].

In a natural population, sexual ornamentation may evolve rapidly, often due to reasons other than sexual selection [8]. Changes in the absolute expression of ornamentation with time [28], [29] lead to changes in the position of preferred trait values in the phenotypic frequency distribution of ornamentation so that the adjustment of preferences is necessary. However, changes in the information content of sexual signals with time due to environmental [30], [31] or genetic reasons [32] may also require evolution in preferences or otherwise the fitness of offspring will be reduced [33]. Finally, signal expression and preferences may evolve together in an arms race [34]. Genetic variation in mate preferences may sometimes maintain genetic variation in signals, although the overall selection is usually still directional (but see [35]). Note that these arguments concern continuously distributed ornamental traits, while discrete polymorphisms raise different questions about selection and mate choice [36], [37].

In spite of its importance, among-individual variation in mate preferences is much less well studied than variation in ornaments [38]. This is partly because mate preference has several often independent attributes such as responsiveness, selectivity and preferred trait value, and these may be difficult to disentangle [39]. Moreover, it is usually logistically difficult or impossible to follow mate sampling in the field, and the intrinsic preferences of individuals may not be expressed due to environmental constraints (i.e. a deviation of mate choice from mate preference) [40]. Therefore, almost all data on individual variation in mate preferences come from laboratory studies, predominantly in insects [41], [42], [43] (but see [44] for a field study), and the same is true for the heritability of preferences [39], [45], [46], [47].

To the best of our knowledge, there are only two studies of the inheritance of mating patterns in the wild. Both studies used the ornamentation of the mate as a measure of an individual's mate preference. In the first study [48] barn owl (*Tyto alba*) fathers and sons were found to positively correlate in the ornamentation of females they mated with, and mate ornamentation was also repeatable within males. In this study, additive genetic and other individual-specific (i.e. permanent environment) effects on mate attractiveness could not be distinguished. The second study was conducted in a Swedish population of collared flycatchers (*Ficedula albicollis*) on the island of Gotland, using an extensive, long-term pedigree [49]. When separated from many other variance components, additive genetic variance was high for the ornament itself (forehead patch size, FPS treated as a male trait) but very low for female preference (mate FPS treated as a female trait). Moreover, the genetic correlation between female preference and male ornament was close to zero, so the authors concluded that selection on male FPS could not drive the evolution of female preferences in this population. Critics of this study argued that using mate ornamentation (i.e. actual mate choice) as a preference measure disregards the fact that females cannot always get what they want. In other words, the set of mates available to a female is limited, and female sampling is also limited, so raw mate ornamentation will be loaded with so much environmental noise that a heritability value close to zero is almost inevitable [50]. Given the difficulty of measuring mate preferences in the wild, a potential way forward is to examine factors that may influence estimates of heritability in female mate ornamentation. In the present study, we use data from a Central-European population of collared flycatchers to examine one such background factor.

Studies in many species have suggested that mate choice is age-dependent [51]. This may often be related to the effects of breeding experience on mate sampling. In migratory passerines, breeding success usually declines with date [52], especially in the face of rapid climate change [53]. Breeding experience makes it easier for females to choose an appropriate territory, as demonstrated in our study species [54]. This implies that experienced females will have more time for mate sampling. However, experience may also facilitate discrimination among potential mates [55]. Therefore, we would expect that genetically coded mate preferences will be expressed less strongly in naïve than in experienced females. In a short-lived species such as the collared flycatcher, a large percentage of breeding females is inexperienced [56], which could seriously reduce the apparent heritability of female choice as measured by the proxy of mate attractiveness. Testing this argument could be difficult in situations where male attractiveness is confounded with territory quality [57], but this does not seem to apply to male FPS in the population considered here [58].

Here we concentrate on the effect of breeding experience on the heritability of mate ornamentation. As a first step, we look for effects of breeding experience on breeding date and clutch size when controlling for female age, to see whether our coding of experience is meaningful. As a second step, we repeat the population-level heritability analyses conducted in the Swedish population to see whether the apparent additive genetic background of mate FPS is similar in our population. For comparison, we also estimate the variance components of FPS in males (i.e. using male FPS as a male and not a female trait) [32]. Third, we compare mate ornamentation and the variance components of mate ornamentation between inexperienced and experienced females.

We ignore other sexual traits such as wing patch size and song throughout, due to the lack of adequate data from those traits for this analysis. However, the independent treatment of FPS is justified by the fact that this ornament, with its low phenotypic plasticity, occupies a special, disjunct position among male sexual traits in our population [59], [60]. Our analyses also assume that FPS plays a role in female choice in our population. Three lines of evidence support this. First, large-patched males breed earlier than small-patched males in almost every year [32]. Second, breeding date advantages related to FPS are stronger in years when FPS is relatively larger at the population level compared to the multi-year average than the other white ornament, wing patch size, and the most straightforward explanation to this pattern is sensory bias in the mate choice of females [59]. Third, males with larger FPS also acquire mates sooner after their arrival from migration than small-patched males [60].

## Results

### Breeding experience and breeding parameters

**Figure 1:**
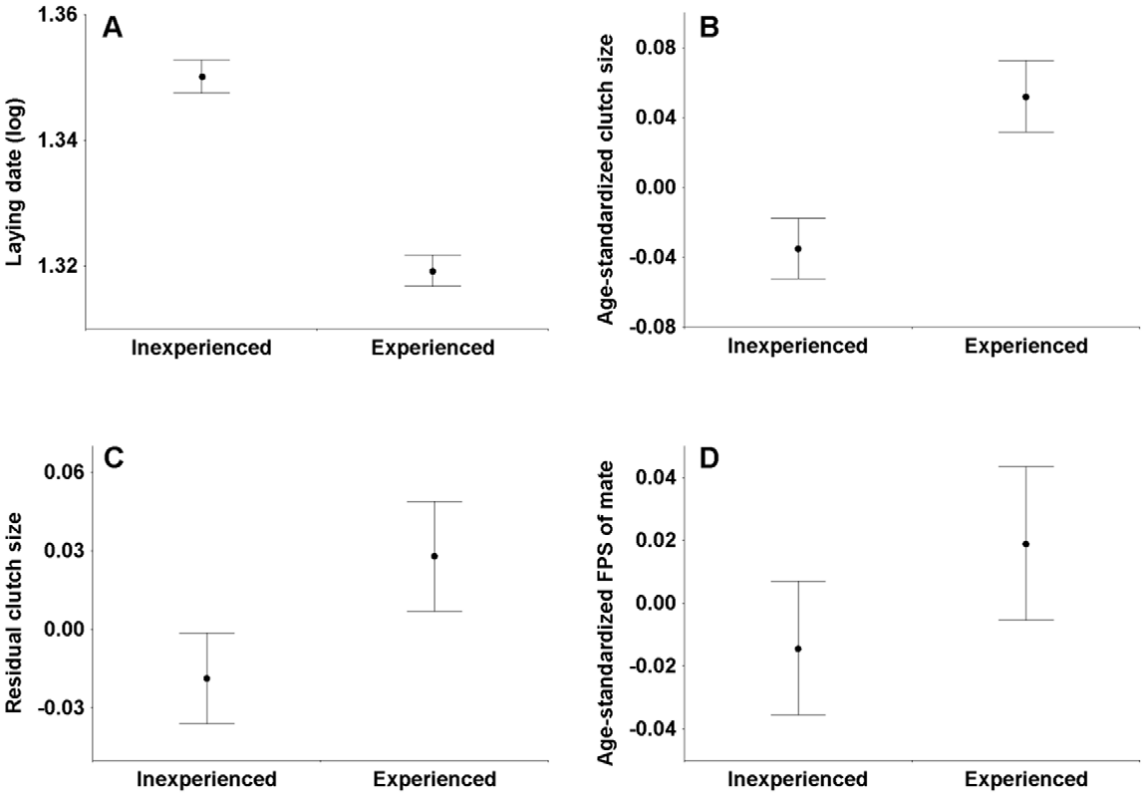
The effect of prior breeding experience on breeding parameters and mate attractiveness in female collared flycatchers. A) laying date (relative to the yearly median, converted to positive, log-transformed); B) clutch size (standardized for binary female age); C) date-residual clutch size (from least squares linear regression, standardized for binary female age); D) forehead patch size (FPS) of mate (standardized for binary male age).

Breeding date (n = 5651 observations) was strongly influenced by experience (F_1,4078_ = 86.57, p<0.001), but less strongly affected by age (F_1,5622_ = 2.90, p = 0.089). Inexperienced females laid eggs much later than experienced ones (Fig. 1A). Clutch size (n = 5611 observations) increased with both experience (Fig. 1B; F_1,5458_ = 21.77, p<0.001) and age (F_1,5604_ = 17.55, p<0.001). The same was true for date-corrected clutch size (Fig. 1C; breeding date F_1,5562_ = 2880.93, p<0.001; experience F_1,5385_ = 7.24, p<0.001; age F_1,5551_ = 17.34, p<0.001).

### Heritability of male FPS and female mate FPS

Animal model analyses for FPS as a male trait are detailed in Table 1. After accounting for the highly significant effect of age, approximately fifty percent of variation in male FPS was explained by the highly significant additive genetic effect (V_A_), while variances due to year (V_YEAR_) and nestbox plot (V_PLOT_) were also significant and together explained another ten percent of variation. V_PE_ was not significant. When analyzing female mate FPS (Table 2), on the other hand, V_PE_ was estimated as zero, while V_A_ was small and not significantly different from zero. The relatively broad error range of the V_A_ estimate implies that its exact value remains uncertain, but it is unlikely to fall above 0.1. Female breeding experience as a fixed effect was non-significant on mate FPS (Fig. 1D), but the random effects of year and nestbox plot were highly significant. Estimating the additive genetic correlation between the ornament and the preference measure (see [49], details not shown) yielded an estimate of 0.293±0.316, which is not statistically different from either 0 or 1, so we do not discuss it further.

**Table 1:** Animal model variance component analysis of forehead patch size in collared flycatcher males.

Variance component	Variance	SE	Ratio to V_P_		SE	LRT χ^2^	p	Fixed effect	F		df	p	r	CIL	CIU
Additive genetic	118.184	16.036	0.497	0.067		74.90	<0.001	Intercept	2041.48	1, 12.9		<0.001			
Permanent environment	16.446	12.651	0.069	0.053		1.76	0.185	Binary age	20.51	1, 1836.3		<0.001	0.105	0.063	0.147
Nestbox plot	13.848	8.765	0.058	0.037		19.94	<0.001								
Year	9.872	4.046	0.042	0.017		35.88	<0.001								
Residual	79.508	3.556	0.334	0.015											

Likelihood ratio tests (LRT) of random effects refer to removal from the full model. The significance of fixed effects was tested with conditional Wald F tests in ASReml. r, effect size (Pearson r); CIL, lower 95% confidence interval; CIU, upper 95% confidence interval.

**Table 2:** Animal model variance component analysis of mate forehead patch size in collared flycatcher females.

Variance component	Variance	SE	Ratio to V_P_	SE	LRT χ^2^	p	Fixed effect	F	df	p	r	CIL	CIU
Additive genetic	9.490	5.243	0.042	0.023	2.14	0.144	Intercept	3132.88	1, 21.5	0.003			
Permanent environment	0.000	-	0.000	-	-	-	Binary mate age	15.83	1, 1959.9	<0.001	0.090	0.046	0.133
Nestbox plot	3.756	2.782	0.016	0.012	11.46	<0.001	Binary experience	2.43	1, 1886.1	0.120	0.036	-0.008	0.080
Year	17.156	6.523	0.075	0.029	77.58	<0.001							
Residual	197.978	7.957	0.867	0.035									

Likelihood ratio tests (LRT) of random effects refer to removal from the full model. The significance of fixed effects was tested with conditional Wald F tests in ASReml. The permanent environment effect was bound to zero and its SE could not be estimated. r, effect size (Pearson r); CIL, lower 95% confidence interval; CIU, upper 95% confidence interval.

### Breeding experience and the proximate determination of female mate FPS

Our final analysis was a bivariate animal model of mate FPS among inexperienced and experienced females. Similar to the univariate results, V_PE_ in the experienced group was fixed at zero. The estimated genetic correlation between experience categories was positive and very close to unity (r_G_ = 0.931±0.940, LRT χ^2^ = 0.00, df = 1, p = 1.0), but it had a broad error range and therefore did not differ significantly from zero either (LRT χ^2^ = 2.46, df = 1, p = 0.117). In this bivariate model, V_A_ seemed slightly higher in the inexperienced group, but it was not significantly different from zero in either category (inexperienced V_A_ = 0.098±0.084, LRT χ^2^ = 1.82, df = 1, p = 0.177; experienced V_A_ = 0.025±0.036, LRT χ^2^ = 0.38, df = 1, p = 0.538). Simultaneously constraining r_G_ as 1.0 and V_A_ in the two experience categories as equal did not lead to a significantly different model likelihood compared to the unconstrained model (χ^2^ = 0.70, df = 1, p = 0.403). In sum, there was no evidence that experience affected the additive genetic background of mate FPS. However, the correlation of nestbox plot effects on mate FPS between experience categories (r_PLOT_) was bound to 1.0 while the correlation of year effects (r_YEAR_) was 0.921±0.098. Both r_PLOT_ and r_YEAR_ were significantly different from zero (LRT r_PLOT_ χ^2^ = 5.18, df = 1, p = 0.023; r_YEAR_ χ^2^ = 14.68, df = 1, p<0.001).

## Discussion

We detected ample additive genetic variation (V_A_) for ornamentation (FPS) as a male trait (fifty percent). For mate FPS as a female trait, however, V_A_ was very low (less than five percent), and breeding experience had no demonstrable effect of on the expression of additive genetic variation. We also found significant spatial and temporal constraints on both the expression of mate FPS and its pattern with experience, and a large percentage of unexplained variance in mate FPS. These results raise questions about the adequate quantification of mate preferences in wild populations.

Studies of mate preferences in birds consist mostly of aviary trials (but see [44], [61]). Even among these, repeated tests of the same female are very rare [62]. Mate preferences are generally difficult to quantify, so their variation is much less well documented than ornament variation [38], [63]. The difficulties have two main reasons. First, actual environmental conditions, physiological state and social settings virtually never permit the full expression of mate preferences at mate choice [18], [64]. Second, variation in mate preferences has at least three levels: responsiveness, discrimination and the preference function. The three levels may be partly independent and they collectively determine the observed outcome [39], [46].

In a wild bird population, even the tracking of sampling behavior itself creates enormous logistic difficulties (e.g. [44]), so the comprehensive assessment of individual preference variation [65] is obviously not possible. Laboratory mate choice trials, on the other hand, may not reflect the natural sampling situation and may therefore produce various biases [65] and give divergent results according to experimental design [66]. Moreover, such experiments cannot be conducted on wild birds in numbers that would allow quantitative genetic analyses. Therefore, the genetic background of female preferences remains elusive, and the improvement of estimates from the wild is an important goal. These estimates will almost always be based on mate ornamentation as a measure of preference [48], [49].

The ornamentation of the current mate is determined partly by female preference, but also by sampling strategy and environmental factors such as mate availability, average male quality and female condition [65]. In case of hole-breeding birds such as flycatchers, nest site limitation [67] may drastically suppress male density [68] and thereby limit mate sampling, especially if male attributes vary non-randomly in space. Females may therefore end up competing for breeding opportunities [69]. If current mate ornamentation is largely determined by intrinsic and environmental constraints, it will very poorly reflect individual preferences [70]. In this case, the repeatability and additive genetic variation of mate ornamentation should remain low (as found in [49]) irrespective of population, dataset size and environment, and this will also limit the value of genetic correlations and estimates of indirect selection on preferences [50]. In support of this, we found a low and non-significant heritability in mate attractiveness that was numerically similar to that reported previously in the Swedish population [49]. Powerful animal model estimation of V_A_ and V_PE_ requires adequate dataset size and pedigree depth [71], but this is unlikely to be a limiting factor in our case since analyzing FPS as a male trait revealed a highly significant additive genetic component (similar to previous estimates, see [32], [72]).

If we accept that mate ornamentation can be a problematic measure of female preference, how could we advance our understanding of preferences in the wild when we are still restricted to use this preference measure? One possible solution is to minimize the effect of confounding factors. Here we explored breeding experience as a potential non-genetic confounder of the quantitative genetics of mate attractiveness variation in wild populations. Mate choice among inexperienced females is constrained mainly by the lack of time for sampling due to late arrival (see also [73]), but problems may also arise with territory choice or mate discrimination.

We coded breeding experience relative to our own breeding dataset. It was therefore possible that some old but seemingly inexperienced females had already bred outside the study plots. To see how much this may have reduced the apparent phenotypic effects of breeding experience, we compared the experienced and inexperienced categories for two basic breeding measures, breeding date and clutch size, which were expected to strongly improve with experience (e.g. [74], [75], [76]). When controlling for age, we found earlier breeding, larger clutches, and larger date-corrected clutch size in the experienced group than in the inexperienced group. These apparent experience effects could be completely explained by female condition or genotype if females with later ages of first breeding were consistently better in these respects [56], [77], but this did not seem to be the case (results not shown). Therefore, we could reasonably expect an increased expression of additive genetic variation in mate preferences with breeding experience. However, we found a non-significant opposite pattern. This result has two competing explanations. First, the lack of experience may not constrain the expression of innate preferences, but we consider this unlikely. Temporal constraints on mating are most likely present in the inexperienced group because their pronouncedly later breeding (this study) is more likely to reflect late arrival than a longer mate sampling period (see also [70]), and later arriving females probably devote less time to mate sampling [78]. Moreover, an experiment suggested that learning processes are important in shaping mate preferences in collared flycatchers [79]. The other explanation for the lack of experience effect on mate FPS heritability is that mate FPS is not a good preference measure. But how strong is the likely correspondence between mate FPS and mate preferences?

The overall V_A_ of mate FPS was very low in our population (see also [49]). Moreover, after correcting for mate age, V_A_, V_PE_, V_PLOT_ and V_YEAR_ together accounted for less than 15% of variation in mate FPS in the pedigree analysis. In contrast, laboratory studies using more direct mate preference measures detected robust repeatability, additive genetic and permanent environment effects [46], [47], [62]. Mate attractiveness reflects female preferences only if females can sample adequate numbers of males of varying ornament size so that they can “get what they want” [50]. In nature, however, female choice is limited by many factors that may also cause large amounts of unexplained variance in mate ornamentation.

These limiting factors include temporal changes in the ornamentation of available males [8], within-season changes in male availability and spatial patterns in male attractiveness [40], limited sampling due to competition for mates [69], and female quality effects [80]. In our population, all of these limitations seem to be present. Average male FPS strongly changed during the study period, most likely due to genetic reasons [32]. Furthermore, spatial autocorrelation in male ornamentation has been shown to affect the interpretability of female mating patterns [58] and female quality and competition also seem to limit settlement patterns [81], [82]. The limitation of mate attractiveness by temporal and spatial constraints was clearly visible in our results. Year and nestbox plot effects on mate FPS were highly significant in all analyses, indicating the non-random distribution of available males in time and space. However, year and nestbox plot effects on mate FPS were also significantly correlated between female experience categories (r_PLOT_ and r_YEAR_ respectively). Therefore, if an inexperienced and an experienced female shared breeding area or year, this made their mate FPS similar. At the population level, the numbers of available data varied vastly among combinations of year and nestbox plot, so the high r_PLOT_ and r_YEAR_ we found also imply that temporal and spatial heterogeneity tended to blur any existing experience effect on mate FPS. In other words, temporal and spatial constraints not only affect the distribution of mate FPS, but may also limit the detectability of other, functionally independent, biologically meaningful effects on this trait.

Therefore, mate ornamentation seems to be a poor measure of mate preferences in our population, and this makes its heritability and patterns with experience difficult to interpret. In response to a similar critique [50] of their paper, the authors of Ref. [49] suggested [83] that mate ornamentation may still be meaningful if viewed as a measure of mate choice (and not preference) because selection in the wild acts on actual mate choice and not on intrinsic mate preferences. However, the potential individual variation in mate sampling, the large temporal and spatial heterogeneity in mate availability and ornamentation, and breeding site fidelity together imply that mate ornamentation may also be a bad measure of mate choice. Mate ornamentation would correctly measure the mate choice decision made by a female if the choices of all females were made from the same overall pool, but this is clearly unlikely. This uncertainty may largely cause the patterns we reported above for mate FPS.

To summarize, it seems that the limiting factor in the correlative approach introduced by Ref. [49] is not a single confounding variable such as breeding experience, but the large amount of variance introduced by using mate attractiveness as a mate preference measure (see also [50]). In the collared flycatcher, this variance cannot be efficiently reduced by correcting for study area and year effects. Solutions to this problem may include the use of various female quality measures (condition, body size, ornamentation) as covariates when evaluating mate attractiveness and its heritability [18]. Alternatively, microgeographic, seasonal and female quality constraints may be simultaneously reduced if one can analyze the changes of mate attractiveness within individual females (see [58]), e.g. in random regression animal models [84], but this requires very large datasets. In any case, establishing a more powerful technique to measure mate preference heritability is very important for clarifying the evolutionary role of mate choice in the wild [85], [86]. However, our results clearly indicate that, in contrast to laboratory situations, it will be difficult for females in the wild to be repeatable in their mate choice, so field and laboratory approaches to mate choice should play complementary roles in the future.

## Materials and Methods

### Ethics statement

All work was conducted with ringing license from the Hungarian Ornithological and Nature Conservation Society (MME, registration number 128), long-term research agreements with the Pilis Park Forestry (December 1988 and March 2007) and research permits from Duna-Ipoly National Park and the regional nature conservation authority (DINP 3295/2/1998, DINP 1255/2/2001, DINP 2256-3/2002, DINP 1931-2/2003, DINP 2573/2/2004, KTVF 15951/2005, KTVF 22021/2006, KTVF 16360-2/2007, KTVF 43355-1/2008).

### Field procedures

The fieldwork was conducted in the Pilis Mountains, near Szentendre, Hungary, where a nestbox breeding population (approx. 800 nestboxes, approx. 300 breeding pairs per year) of collared flycatchers has been intensively monitored since 1982. More details on the population and the study site have been presented elsewhere [32], [68], [73]. Nestboxes were regularly checked during the whole breeding season, with more frequent checks in the nest building and egg laying stages. Nestlings were ringed with standard numbered metal rings at 6 to 10 days of age. Parents were usually caught at 8 to 12 days of nestling age, ringed if necessary and standard measurements were taken of morphology and plumage ornamentation. The maximum width and height of male FPS were measured with a caliper, to the nearest 0.1 mm. Males were not captured regularly before 1989, so the present analyses use 21 years of pedigree data from 1989 to 2009 (phenotypic data 1989–2009, recruitment data 1989–2007). Data on male FPS are available from 1990 onwards. Capture effort was consistently high throughout the study period.

In our population, extrapair paternity does not seem to be consistently related to any male trait, including FPS [87], [88] (but see [89]), so extrapair paternity does not seem to represent a mechanism by which females can “correct” their social mating decision as suggested for the Swedish population [50]. In other words, sire attractiveness in our population is largely determined by social mate choice and not by extrapair paternity. Moreover, parent-offspring misassignment due to moderate levels of extrapair paternity does not seem to strongly bias animal model heritability estimates [90].

### Statistical analyses

The comprehensive 21-year phenotypic dataset from which we drew our data contained n = 4233 male FPS records and n = 3726 female mate FPS records. Repeated records of individuals within years and all broods where brood size had been manipulated or the nestlings had been cross-fostered without individual identification were deleted from the analyses. Of the remaining data, those that could be used for the pedigree analyses (recruits and their parents) consisted of n = 2138 male FPS records and n = 1971 female mate FPS records from n = 1380 recruits and their parents (n = 1354 maternal and n = 1205 paternal links).

Data were analyzed using ASReml2 (VSN International) to fit a series of animal models. An animal model is a linear mixed effect model that includes individual genetic merit as a random effect such that, in the presence of pedigree data, phenotypic variance can be partitioned into (additive) genetic and environmental components [71], [91], [92]. Additional effects were also included as described below with the significance of random effects determined using likelihood ratio tests (LRT; comparison of full and reduced models) and fixed effects tested using conditional Wald *F* statistics. Importantly, estimates of additive genetic variance and covariance in an animal model depend on the other random and fixed effects in the model [93]. We also cannot rule out that these estimates include unmeasured environmental variance components that are confounded with the pedigree (e.g. various common environment effects, [94]). Although statistical approaches in the absence of controlled experiments cannot completely eliminate the risk of bias, animal model estimates are expected to be more robust in this respect than classical approaches such as parent-offspring regression [71].

Firstly, we looked for effects of female breeding experience on breeding date (log-transformed first egg date relative to yearly median) and clutch size using general linear mixed models in PROC MIXED of SAS 9.1 (SAS Institute). The effect of breeding experience is confounded by age, so we tested experience effects in two ways: with binary age as a simultaneous predictor, and in the subset of after-second-year (i.e. old) females. The two approaches always gave the same results, so we report results of the former, which relies on all available data and is therefore more powerful. All models contained female identity, nestbox plot (i.e. breeding area) and year as random effects, and female age and experience as fixed factors. For clutch size, we also ran a model with breeding date as a covariate to see whether experience affects primary reproductive output when controlling for differences in breeding date.

Secondly, we estimated variance components for female mate FPS and male FPS. For female mate FPS, the model included binary mate age (yearling or older male) and binary breeding experience (inexperienced or experienced female based on prior breeding on our nestbox plots) as fixed factors. For male FPS, the only fixed factor was the binary age of the male. Male age as a fixed effect was included because FPS is weakly but significantly age-dependent [32]. In both models, the same set of random effects was then used to partition phenotypic variance into additive genetic (V_A_), permanent environment (V_PE_), breeding area (V_PLOT_), year of breeding (V_YEAR_), and residual (V_R_) components. Plot and year effects were included to account for expected spatial and temporal heterogeneity in the environment. The permanent environment effect makes use of repeated measures available from individuals to account for fixed non-genetic differences between individuals that can otherwise bias the estimation of V_A_. Phenotypic variance V_P_ was determined as the sum of the variance components and heritability estimated as the ratio of V_A_ to V_P_. Note that this model is similar to that used by Ref. [49] except for the inclusion of male age and female experience.

Thirdly, to test the null hypothesis that the heritability of mate ornamentation does not change with female experience, we ran a bivariate animal model in which the mate FPS of inexperienced and experienced females were modeled as two separate traits. We used the above model structure except that the permanent environment component was fit only in the experienced group. This is because inexperienced birds were defined as those in their first breeding attempt and therefore there can be no repeated measures of this trait. Heritabilities for each trait were estimated as well as the genetic covariance between them which was rescaled to estimate the genetic correlation r_G_. The area- and year-generated covariances between the experience categories were modeled as well (r_PLOT_ and r_YEAR_, respectively). As an explicit test for a genotype by-breeding experience interaction we compared the likelihood of this model to one in which r_G_ was set to unity and V_A_ was constrained to be constant across experience classes.
